# PD189 Strengthening Health Decision-Making: The Role Of Capacity Building In Health Technology Assessment In Latin America

**DOI:** 10.1017/S0266462324004112

**Published:** 2025-01-07

**Authors:** Carla Colaci, Verónica Andrea Alfie, Sebastian Garcia Marti, Andrea Alcaraz, Federico Augustovski, Andres Pichon-Riviere

## Abstract

**Introduction:**

Health technology assessment (HTA) applications in low- and middle-income countries face limited technical capacities. The Institute for Clinical Effectiveness and Health Policy (IECS) is a key player in strengthening HTA expertise in the region and offers a variety of courses. Over 200 students from across Latin America have undertaken our introductory course on HTA and economic evaluation in the last four years.

**Methods:**

The IECS provides a nine-week introductory online course focused on the fundamentals of HTA and economic evaluation. The course is designed for healthcare professionals (doctors, administrators, auditors, nurses, pharmacists, lawyers, etc.). The materials are available in Spanish and Portuguese on a virtual campus with asynchronous activities. Students are guided by instructors through an exchange forum. This study aimed to showcase the outcomes of this HTA course. Our analysis encompassed quantitative and qualitative data from a survey administered to nine student cohorts over the last four years. The surveys featured eight question categories covering materials, activities, quizzes, course dynamics, forum usage, tutoring, and satisfaction.

**Results:**

A total of 234 students from Latin America were enrolled in the course. More than half came from Argentina (68%). Of the initial enrollees, 212 (91%) started the course and 192 (91%) of them passed. The satisfaction survey was completed by 168 students. Ninety-six percent of students were satisfied or very satisfied with the course overall, and the same percentage would recommend it to a colleague. Eighty-six percent felt that they could adequately follow the course, and 40 percent of students dedicating an average of two to four hours per week to the course.

**Conclusions:**

Having accessible and feasible training opportunities in the region is important. The IECS HTA and economic course enhances HTA expertise in Latin America, as evidenced by its high rates of enrolment, completion, and satisfaction, with over 90 percent of participants recommending it. This underscores its effectiveness in reinforcing health decision-making knowledge in Latin America and contributing to the advancement of health policy.

